# Effects of Maternal 5,10-Methylenetetrahydrofolate Reductase C677T and A1298C Polymorphisms and Tobacco Smoking on Infant Birth Weight in a Japanese Population

**DOI:** 10.2188/jea.JE20110039

**Published:** 2012-03-05

**Authors:** Thamar Ayo Yila, Seiko Sasaki, Chihiro Miyashita, Titilola Serifat Braimoh, Ikuko Kashino, Sumitaka Kobayashi, Emiko Okada, Toshiaki Baba, Eiji Yoshioka, Hisanori Minakami, Toshiaki Endo, Kazuo Sengoku, Reiko Kishi

**Affiliations:** 1 Department of Public Health Sciences, Hokkaido University Graduate School of Medicine, Sapporo, Japan; 2 Department of Obstetrics and Gynecology, Hokkaido University Graduate School of Medicine, Sapporo, Japan; 3 Department of Obstetrics and Gynecology, School of Medicine, Sapporo Medical University, Sapporo, Japan; 4 Department of Obstetrics and Gynecology, School of Medicine, Asahikawa Medical College, Asahikawa, Japan; 5 Center for Environmental and Health Sciences, Hokkaido University, Sapporo, Japan

**Keywords:** birth weight, tobacco smoking, *MTHFR* SNPs, folate, Japan

## Abstract

**Background:**

Intracellular folate hemostasis depends on the 5,10-methylenetetrahydrofolate reductase (*MTHFR*) gene. Because 5,10-*MTHFR* 677TT homozygosity and tobacco smoking are associated with low folate status, we tested the hypothesis that smoking in mothers with 5,10-*MTHFR* C677T or A1298C polymorphisms would be independently associated with lower birth weight among their offspring.

**Methods:**

We assessed 1784 native Japanese mother-child pairs drawn from the ongoing birth cohort of The Hokkaido Study on Environment and Children’s Health. Data (demographic information, hospital birth records, and biological specimens) were extracted from recruitments that took place during the period from February 2003 to March 2006. Maternal serum folate were assayed by chemiluminescent immunoassay, and genotyping of 5,10-*MTHFR* C677T/A1298C polymorphisms was done using a TaqMan allelic discrimination assay.

**Results:**

The prevalence of folate deficiency (<6.8 nmol/L) was 0.3%. The 5,10-*MTHFR* 677CT genotype was independently associated with an increase of 36.40 g (95% CI: 2.60 to 70.30, *P* = 0.035) in mean infant birth weight and an increase of 90.70 g (95% CI: 6.00 to 175.50, *P* = 0.036) among male infants of nonsmokers. Female infants of 677TT homozygous passive smokers were 99.00 g (95% CI: −190.26 to −7.56, *P* = 0.034) lighter. The birth weight of the offspring of smokers with 5,10-*MTHFR* 1298AA homozygosity was lower by 107.00 g (95% CI: −180.00 to −33.90, *P* = 0.004).

**Conclusions:**

The results suggest that, in this population, maternal 5,10-*MTHFR* C677T polymorphism, but not the 5,10-*MTHFR* A1298C variant, is independently associated with improvement in infant birth weight, especially among nonsmokers. However, 5,10-*MTHFR* 1298AA might be associated with folate impairment and could interact with tobacco smoke to further decrease birth weight.

## INTRODUCTION

Intracellular folate hemostasis depends on the 5,10-methylenetetrahydrofolate reductase (*MTHFR*) gene, which is located at position 36 on the short arm of chromosome 1. This gene codes for the enzyme *MTHFR*, which catalyses the irreversible conversion of 5,10-*MTHFR* to 5-metyltetrahydrofolate, a substrate for methylation of homocysteine to methionine. Thus far, 14 rare mutations in *MTHFR* have been described, but the 2 most common single nucleotide polymorphisms (SNPs) are 5,10-*MTHFR* C677T (dbSNP ID: rs1801133)—a missense mutation in exon 4, characterized by an alanine to valine substitution on codon 222—and 5,10-*MTHFR* A1298C (dbSNP ID: rs1801131)—a point mutation in exon 7 characterized by a glutamate to alanine substitution on codon 429.^1^ 5,10-*MTHFR* C677T is located in the catalytic N-terminal domain of the enzyme, while 5,10-*MTHFR* A1298C is located in the regulatory domain of the enzyme.^2^

Biochemically, the 5,10-*MTHFR* C677T polymorphism is associated with thermolability and reduced enzyme activity. The metabolic consequences are folate deficiency and mild hyperhomocysteinemia, a risk factor for thrombotic vascular diseases. It has been suggested that oxidative stress, platelet aggregation, and endothelial cell dysfunction contribute to the vasculotoxicity of homocysteine, and 5,10-*MTHFR* polymorphisms have been widely investigated in relation to a spectrum of several disease outcomes. Specifically, several studies have identified maternal 5,10-*MTHFR* C677T polymorphisms as obstetric genetic risk factors for spina bifida, placenta-related vasculopathies, spontaneous fetal loss, preterm delivery (PTD), low birth weight (LBW), small for gestational age (SGA), neurodevelopmental delays, and other congenital anomalies.^3^^–^^15^ However, several other investigators have found no such associations.^16^^–^^25^ This confusion might be explained by the fact that phenotypic expression of this genetic trait depends on folate status and other environmental factors that vary by geographic region and race.

Although the functional consequences of the 5,10-*MTHFR* A1298C variant are not well known, it is a risk factor for neural tube defects,^26^^,^^27^ and compound heterozygosity (5,10-*MTHFR* 677CT/1298AC) has been reported to have a biochemical profile similar to that of 677TT homozygosity.^1^^,^^28^

Maternal smoking during pregnancy is an established risk factor for intrauterine growth retardation (IUGR), SGA, PTD, and other adverse pregnancy outcomes.^29^ More recently, smoking has been associated with nutritional deficiencies, including folate deficiency.^30^^–^^32^ LBW secondary to IUGR or PTD remains a public health concern because it increases the risk of morbidity and mortality throughout life.

In Japan, there have been genetic association studies of the relation between folate and cardiovascular pathologies, cancers, *Helicobacter pylori* infection, and periodontal diseases. However, only a few such studies have investigated obstetric events, and none has considered infant birth size.^3^^,^^6^^,^^17^^,^^33^^–^^35^ We therefore tested the hypothesis that maternal smoking in the presence of the 5,10-*MTHFR* C677T or A1298C polymorphisms independently reduces birth weight.

## METHODS

### Study design and participants

**Figure 1.**
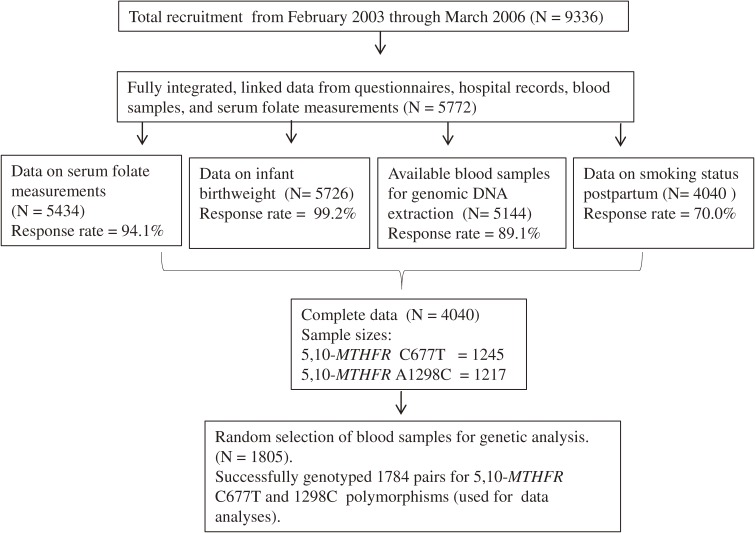
Study selection flow chart

The study participants were native Japanese mother-child pairs drawn from an ongoing birth cohort: The Hokkaido Study on Environment and Children’s Health. This ongoing cohort started in February 2003, and the details of the study have been previously described.^36^ Briefly, all indigenous Japanese women who reserved antenatal care at any of 37 participating hospitals within Hokkaido during their first trimester of pregnancy were considered eligible. Health care personnel introduced the study, after which each potential participant was given an invitation that included a consent form, baseline questionnaire, and self-addressed envelopes for return of the signed consent forms and completed questionnaires. The participants were recruited between February 2003 and March 2006. Only participants with linked and integrated data (5772; 61.8%) were included in the baseline population of this study. The response rate for each variable was at least 70.0% from various sources. Based on the population allele frequencies of the 5,10-*MTHFR* C677T^37^ and A1298C^38^ polymorphic variants specific to Japanese and the prevalence of tobacco smoking during pregnancy,^39^ minimum sample sizes were calculated by using genetic software.^40^ We randomly selected 1805 extracted genomic DNAs, attempted to discriminate the alleles of 5,10-*MTHFR* polymorphisms, and successfully genotyped 1784, which were ultimately used in the data analysis (Figure 1). The Institutional Ethical Board for Human Gene and Genome Studies of Hokkaido University Graduate School of Medicine approved the study protocol.

### Methods of data collection

Data were acquired from baseline self-administered questionnaires, hospital records of infant births, and postpartum self-administered questionnaires. Baseline information included biodata, lifestyle habits, drugs (including use of nutritional supplements), and gynecologic and obstetric histories. Infant birth records from hospitals had information about birth weight, gestational age at delivery, sex, obstetric events during index pregnancy, and congenital anomalies, among other information. Postpartum questionnaires collected information on infant anthropometric parameters, active or passive tobacco smoking during the index pregnancy, and whether the index pregnancy was eventful. Each variable in the dataset had a response rate of at least 70.0%, although the item on smoking status decreased from 99.0% at baseline to 70.0% after pregnancy. Whole-blood specimens were collected during the first trimester for serum folate assays; subsequent collections of whole blood specimens were stored at −80°C for genetic analyses.

### Folate assay

Serum folate was assayed by a commercial laboratory (SRL, Inc. Tokyo, Japan) using an automated competitive protein binding (CPB) chemiluminescent enzyme immunoassay (CLEIA) technique according to the manufacturer’s protocols. This type of assay has an intra- and inter-assay imprecision of 10.0% or less and has become common in in vitro studies, as it is less costly, faster, and convenient. In addition, the need for smaller samples is advantageous in large scale epidemiologic studies.^41^ The specific assay method for this study was the ADVIA Centaur technique, which has a coefficient of variation between 4.0% to 4.3%.^42^ Analyses were conducted in batches, which were scheduled with regard to recruitment period and laboratory procedure.

### Selection and genotyping of single nucleotide polymorphisms (SNPs)

We chose the 2 most common SNPs of this gene, namely C677T and A1298C, which have minor allele frequencies (MAF) of 35.2%^37^ and 19.0%,^38^ respectively, among Japanese. Genomic DNAs were extracted using a Maxwell 16 Instrument (Promega Corporation, WI, USA). DNA amplifications were performed in batches on 96-well micro-amp reaction plates using validated TaqMan probes for *MTHFR* C677T and A1298C (assay IDs: C_1202883_20 and C_850486_20), respectively, on a Gene Amp 9700 thermal cycler (Applied Biosystems, Foster City, CA, USA) with an end-point allelic discrimination (AD) assay on a 7300/7500 Real-time PCR System^43^ (Applied Biosystems, Foster City, CA, USA). We randomly selected 95 samples (5.0% of the successfully genotyped samples) and repeated genotyping to check for genotyping quality. The results were 100% concordant.

### Definition of variables

#### Environmental exposures

Overall smoking status was classified into 3 categories using both self-reported active smoking and passive exposure to environmental tobacco smoke (ETS) at home. Nonsmokers had no history of active smoking or exposure to ETS at home. Nonsmokers and quitters with ETS exposure were classified as the passive smoking group, while smokers consisted of active smokers irrespective of ETS exposure status. Quitters with no ETS exposure during the first trimester had mean infant birth weights similar to those of nonsmokers; hence, they were added to the nonsmoking group. Mothers who quit during the second or third trimesters were added to the active smoking group.

#### Genetic exposures

The 5,10-*MTHFR* C677T and A1298C genotypes were categorized as dominant homozygous, heterozygous, and recessive homozygous genotypes (677CC, 677CT, and 677TT; and 1298AA, 1298AC, and 1298CC, respectively).

### Statistical analyses

Univariate ANOVA with multiple comparison tests was performed to assess the main effects of maternal 5,10-*MTHFR* C677T and A1298C polymorphisms and smoking on serum folate levels, while ANCOVA was used to investigate the interactive association between smoking and 5,10-*MTHFR* C677T and A1298C polymorphisms in relation to folate status and infant birth weight. Serum folate was log-transformed before the analyses and back-transformed after the analyses. Known major predictors of infant birth weight (infant sex, gestational age at delivery, maternal age, maternal prepregnancy weight, maternal height, parity, and alcohol intake during pregnancy) were adjusted for in the multivariate regression analyses. Use of a folic acid supplement, which was highly correlated with serum folate levels, was also included as a covariate. Smoking status was adjusted for when we assessed the predictive power of each SNP on birth weight. Categorical covariates were dichotomized to fit the regression equation. Subgroups with few participants (ie, subgroups for the 1298CC genotype) were excluded from the regression analyses. We used the codominant genetic model and per-allele approach. Our preliminary analyses revealed that mean serum folate was highest for the *MTHFR* 1298AC genotype. Because adequate folate status is an integral part of our hypothesis, we decided that it was biologically plausible to set 1298AC as the reference category in the regression analyses. Predictors were entered simultaneously into the equation. Assessment of the 5,10-*MTHFR* C677T and A1298C genotypes for deviation from the Hardy-Weinberg equilibrium, and other evaluations of data quality, were conducted using Haploview version 4.2 software.^44^ All other analyses were performed using SPSS version 16.00 for Windows (SPSS Inc., Chicago, IL, USA). The level of statistical significance was set at less than 0.05.

## RESULTS

**Figure 2.**
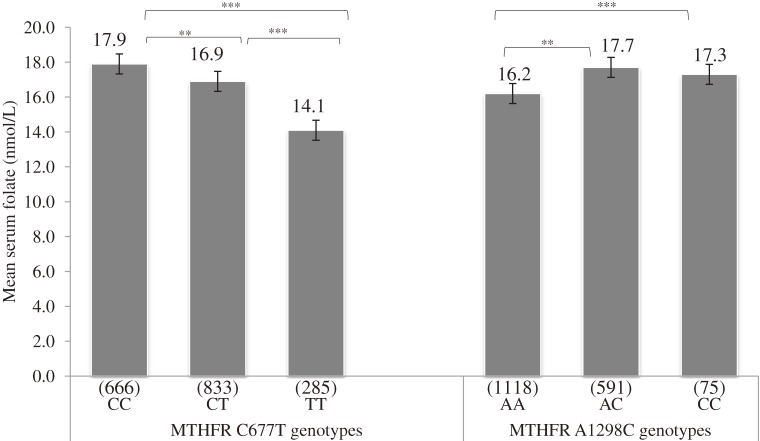
Maternal mean serum folate levels across *MTHFR* C677T and A1298C genotypes.  **P < 0.01, ***P < 0.001 Univariate analysis with Tukey’s honestly significant differences test. Values in parentheses are counts in each group.

**Figure 3.**
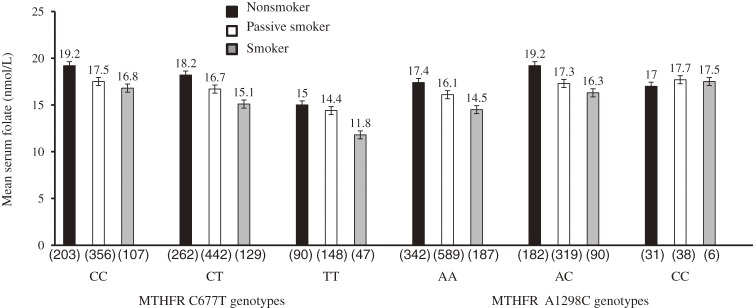
Maternal mean serum folate levels across *MTHFR* C677T and A1298C genotypes according to smoking status.  ANCOVA *P*_interaction_ < 0.001. For MTHFR C677T and A1298C. Values in parentheses are counts in each group.

The maternal mean serum folate (SD) level was 16.4 (1.5) nmol/L. The prevalence of folate deficiency (<6.8 nmol/L) was 0.3%; most (73.0%) mothers had adequate folate status (≥13.6 nmol/L). The prevalence of folic acid supplementation was 10.0%. Mean infant birth weight (SD) was 3040 (374) g. The prevalence of active smoking during pregnancy was 15.9%, while that of passive smoking was 53.0%. The distributions of the 5,10-*MTHFR* C677T and A1298C genotypes did not deviate from the Hardy-Weinberg equilibrium (*P* = 0.546 and 0.909, respectively). The frequencies of *MTHFR* 677CC, 677CT, and 677TT were 37.3%, 46.7%, and 16.0%, respectively, while those of *MTHFR* 1298AA, 1298AC, and 1298CC were 62.7%, 33.1%, and 4.2%, respectively. A strong LD (D′ = 0.943) between *MTHFR* C677T and A1298C was also observed, and the minor allele frequencies were 0.392 and 0.205, respectively (Table 1). These findings were similar to those of previous studies of Japanese populations.^6^^,^^37^^,^^38^^,^^45^^–^^47^ We used 2-way analysis of variance to assess the main effects of 5,10-*MTHFR* on maternal mean serum folate concentration. Carrying the T allele was associated with a decrease in mean serum folate level, and the lowest level (14.1 nmol/L) was observed in the 677TT homozygous group. Tukey’s honestly significant differences (HSD) of 1.0 nmol/L (*P* = 0.008) and 3.8 nmol/L (*P* < 0.001) were observed between 677CC versus 677CT and between 677CC versus 677TT, respectively. In contrast, carrying the 1298C allele was associated with higher mean serum folate levels. A Tukey’s HSD of 1.5 nmol/L (*P* < 0.001) was observed between 1298AA versus 1298AC (Figure 2). Mean serum folate levels in the analysis of covariance were generally lower among smokers for all 5,10-*MTHFR* C677T genotypes, and the lowest level was found among 677TT homozygotes (11.8 nmol/L, *P*_interaction_ < 0.001). With regard to 5,10-*MTHFR* A1298C genotypes, the lowest mean folate level was observed among smokers with the 1298AA homozygous genotype, (*P*_interaction_ < 0.001; Figure 3).

**Table 1.** Characteristics of 1784 mother-child pairs

Characteristic	*n* (%)
Maternal age (years)	30.0 (4.3)^e^
Maternal height (cm)	158.0 (5.1)^e^
Prepregnancy weight (kg)	53.0 (9.3)^e^
Maternal serum folate (nmol/L)	16.4 (1.5)^e^
Gestational age at delivery (weeks)	38.9 (1.3)^e^
Infant birth weight (g)	3040 (374)^e^
Infant sex	
Male	873 (48.9)
Female	911 (51.1)
Parity	
Nulliparous	391 (21.9)
Parous	1393 (78.1)
Alcohol intake during pregnancy	
No	1499 (84.0)
Yes	285 (16.0)
Tobacco smoking during pregnancy	
Nonsmoker	555 (31.1)
Passive smoker	946 (53.0)
Smoker	283 (15.9)
Folic acid supplementation	
No	1601 (89.7)
Yes	183 (10.3)
Maternal *MTHFR* C677T genotype^a,b^	
CC	666 (37.3)
CT	833 (46.7)
TT	285 (16.0)
CT/TT	1118 (62.7)
Maternal *MTHFR* A1298C genotype^c,d^	
AA	1118 (62.7)
AC	591 (33.1)
CC	75 (4.2)
AC/CC	666 (37.3)

^a^HWE = Hardy-Weinberg equilibrium *P* = 0.5463

^b^MAF = Minor allele frequency = 0.392

^c^HWE = Hardy-Weinberg equilibrium *P* = 0.9091

^d^MAF = Minor allele frequency = 0.205

^e^Mean (SD) *MTHFR* = Methylenetetrahydrofolate reductase gene

We initially explored the role of maternal serum folate during the first trimester as a predictor of infant birth weight and found no significant linear association. After stratification by folate status, low folate status (<13.6 nmol/L) was associated with a nonsignificant reduction in birth weight. After stratification by birth weight status, low folate status was associated with a 34.00 g (*P* = 0.045) reduction in mean birth weight of infants in the normal birth weight group (data not shown).

To investigate whether the maternal 5,10-*MTHFR* C677T and A1298C polymorphisms were independently associated with birth weight, we conducted a multiple regression analysis with adjustments for known major predictors of birth weight. Among infants of 677CT heterozygous mothers, adjusted mean birth weight was highest (3061 g) for 5,10-*MTHFR* C677T. The 677CT genotype was associated with a 36.40 g increase in mean infant birth weight (95% CI: 2.60 to 70.30, *P* = 0.035). Carrying the 677T allele was associated with a marginally significant 27.00 g increase in infant birth weight (95% CI: −3.76 to −59.47, *P* = 0.084).

Polymorphism in 5,10-*MTHFR* A1298C or carrying the 1298C allele was not significantly independently associated with birth weight, although the adjusted mean infant birth weight was highest (3048 g) in the 1298AC heterozygous group. The adjusted mean birth weight of infants of active tobacco smokers was lowest (2978 g) and was 85.00 g (95% CI: 133.30 to −36.80, *P* = 0.001) less than that of children born to nonsmokers (Table 2).

**Table 2.** Association of maternal 5,10-*MTHFR* C677T, A1298C genotypes and tobacco smoking with infant birth weight (*N* = 1784)

Maternal 5,10-*MTHFR* polymorphisms/smoking status	*n*	Adjusted mean birth weight (SE) g	Adjusted Δ B (SE) [95% CI] g	*P*_trend_^c^
^a^*MTHFR* C677T genotype				
CC	666	3024.70 (14.51)	Reference	
CT	833	3061.13 (12.98)	36.40 (17.30) [2.60, 70.30]*	
TT	285	3015.15 (22.75)	4.00 (23.50) [−42.20, 50.10]	0.07
^a^*MTHFR* C677T allele				
C	2165	3044.97 (9.68)	Reference	
T	1403	3049.52 (11.29)	27.86 (16.12) [−3.76, 59.47]^†^	
^a^*MTHFR* A1298C genotype				
AA	1118	3036.58 (11.17)	Reference	
AC	591	3048.71 (15.67)	14.47 (16.78) [−18.45, 47.38]	
CC	75	3028.59 (44.69)	−27.65 (39.36) [−104.85, 49.55]	0.49
^a^*MTHFR* A1298C allele				
A	2827	3040.77 (9.1)	Reference	
C	741	3046.43 (14.78)	9.74 (16.16) [−21.95, 41.43]	
^b^Smoking status				
Nonsmoker	555	3062.81 (14.11)	Reference	
Passive smoker	946	3046.08 (10.70)	−14.40 (17.90) [−49.50, 20.60]	
Smoker	283	2978.29 (19.70)	−85.01 (24.60) [−133.30, −36.80]**	0.001

**P < 0.01. *P < 0.05. ^†^*P* < 0.1. Δ (Change in mean birth weight) B (Unstandardized coefficients) SE (standard error). (CI) confidence interval. (*MTHFR*) Methylenetetrahydrofolate reductase. Grams (g). ^a^Multiple linear regression adjusted for gestational age, infant sex, maternal age, prepregnancy weight, height, parity, smoking during pregnancy, alcohol intake during pregnancy, and folic acid supplement use. ^b^Multiple linear regression adjusted for gestational age, infant sex, maternal age, prepregnancy weight, height, parity, and folic acid supplement intake. ^c^Polynomial univariate analysis.

We stratified mothers by tobacco smoking status. Among nonsmokers, male infants of 677CT genotype mothers were 90.00 g (95% CI: −2.11 to 182.50, *P* = 0.05) heavier than the infants in the reference category, which was marginally statistically significant, while among passive smokers, female infants of 677TT homozygous mothers were 99.00 g (95% CI: −190.26 to −7.56, *P* = 0.03) lighter than reference. None of the minor-allele genotypes among smokers showed any significant effect on infant birth weight as compared with those in the major-allele genotypes (data not shown). Per-allele analyses revealed that carrying the 677T allele was associated with a 68.00 g (95% CI: −121.74 to −15.27, *P* = 0.012, *P*_trend_ = 0.003) reduction in mean birth weight among infants of smokers and an 89.00 g (95% CI: −168.89 to −9.56, *P* = 0.028, *P*_trend_ = 0.018) reduction among female infants (Table 3a). Furthermore, smoking in mothers carrying the 1298A allele was associated with a 92.00 g (95% CI: −144.46 to −40.96, *P* < 0.001, *P*_trend_ = 0.091) reduction in mean birth weight. Males were lighter by 79.00 g (95% CI −150.73 to −8.58, *P* = 0.028, *P*_trend_ = 0.228), while females were lighter by 107.00 g (95% CI: −182.78 to −31.54, *P* = 0.006, *P*_trend_ = 0.112; Table 3b).

**Table 3a.** Association of maternal 5,10-*MTHFR* C677T polymorphism and tobacco smoking with infant birth weight (*N* = 1784)

5,10-*MTHFR*	Smoking status	Overall (*N* = 1784)			Males (*N* = 873)			Females (*N* = 911)		
		
		*n*	Adjusted Mean birth weight (SE) g	Adjusted Δ B (SE) [95% CI] g	*n*	Adjusted Mean birth weight (SE) g	Adjusted Δ B (SE) [95% CI] g	*n*	Adjusted Mean birth weight (SE) g	Adjusted Δ B (SE) [95% CI] g
C677T genotype										
CC	Nonsmoker	203	3008.84 (25.84)	Reference	93	3080.01 (37.75)	Reference	110	2949.95 (34.58)	Reference
	Passive smoker	356	3035.93 (19.85)	9.5 (29.3) [−47.9, 66.9]	187	3037.8 (27.16)	−39.6 (40.5) [−119.2, 39.9]	169	3033.88 (29.16)	59.3 (41.6) [−22.3, 140.8]
	Smoker	107	3017.29 (37.52)	−38.7 (39.8) [−116.8, 39.4]	52	3109.23 (52.51)	−37.7 (55.8) [−147.3, 71.8]	55	2928.76 (51.18)	55.3 (56.5) [−166.2, 55.6]
CT	Nonsmoker	262	3092.43 (24.44)	60.0 (31.0) [−0.9, 120.9]^†^	133	3162.77 (35.43)	90.7 (43.20) [6.0, 175.50]*	129	3020.99 (32.59)	34.1 (44.1) [−52.4, 120.6]
	Passive smoker	442	3068.18 (17.15)	47.5 (28.2) [−7.9, 102.9]	198	3114.72 (24.29)	21.2 (40.3) [−57.8, 100.3]	244	3030.45 (23.78)	62.1 (39.1) [−14.6, 138.8]
	Smoker	129	2973.99 (32.43)	−53.1 (37.8) [−127.2, 21.0]	70	3041.21 (35.16)	−39.8 (51.1) [−140.0, 60.4]	59	2894.24 (55.93)	−63.5 (55.2) [−171.9, 44.9]
TT	Nonsmoker	90	3087.77 (38.76)	59.1 (42.0) [−23.3, 141.4]	43	3123.57 (54.59)	15.1 (58.8) [−100.6, 130.5]	47	3055.09 (55.04)	91.7 (59.0) [−24.0, 207.4]
	Passive smoker	148	2984.38 (31.57)	−14.4 (36.1) [−85.2, 56.3]	74	3045.41 (45.87)	11.1 (50.1) [−87.2, 109.4]	74	2922.5 (42.44)	−39.7 (51.1) [−140.0, 60.6]
	Smoker	47	2974.11 (58.61)	−49.1 (53.9) [−154.8, 56.7]	23	3067.13 (73.23)	−53.7 (74.9) [−200.7, 93.3]	24	2884.96 (88.5)	−48.3 (76.7) [−198.8, 102.2]
	*P*_interaction_			0.03			0.02			0.14
C677T allele^d^										
C	Nonsmoker	465	3059.64 (15.39)	Reference	226	3137.62 (21.58)	Reference	239	2987.47 (21.95)	Reference
	Passive smoker	798	3053.06 (11.64)	−13.37 (19.69) [−51.98, 25.25]	385	3075.26 (56.28)	−60.94 (26.60) [−113.15, −8.73]*	413	3030.17 (16.61)	35.80 (29.20) [−21.51, 93.11]
	Smoker	236	2979.64 (21.57)	−63.45 (33.22) [−128.61, 1.71]^†^	122	3046.22 (29.14)	−57.52 (46.52) [−148.82, 33.78]	114	2909.50 (32.18)	−78.85 (47.97) [−173.00, 15.30]
	*P*_interaction_			0.45			0.75			0.06
T	Nonsmoker	352	3084.10 (17.66)	Reference	176	3159.40 (24.30)	Reference	176	3015.04 (25.68)	Reference
	Passive smoker	590	3055.28 (13.57)	−9.93 (17.12) [−23.65, 43.52]	272	3103.07 (19.34)	12.38 (24.16) [−35.03, 59.79]	318	3009.48 (19.08)	7.75 (24.40) [−40.15, 55.64]
	Smoker	176	2975.05 (24.89)	−68.50 (27.14) [−121.74, −15.27]*	93	3042.31 (33.20)	−48.48 (36.51) [−120.13, 23.18]	83	2909.60 (37.38)	−89.22 (40.59) [−168.89, −9.56]*
	*P*_interaction_			0.34			0.48			0.46

***P < 0.001. *P < 0.05. ^†^*P* < 0.1. Δ (Change in mean birth weight), B (Unstandardized coefficient) SE (Standard error). CI (Confidence interval), g (Grams). ^a^Multiple linear regression adjusted for infant sex, gestational age at delivery, maternal age, maternal height, prepregnancy weight, parity, alcohol intake during pregnancy, and folic acid supplement intake. ^b^Multiple linear regression adjusted for gestational age at delivery, maternal age, maternal height, prepregnancy weight, parity, alcohol intake during pregnancy, and folic acid supplement intake. ^c^Excluded from the regression analyses. ^d^*P*_trend_ by smoking status (C allele = 0.021, Males = 0.045, Females = 0.040 and T allele = 0.003, males = 0.073, Females = 0.018).

**Table 3b.** Association of maternal 5,10-*MTHFR* A1298C polymorphism and tobacco smoking with infant birth weight (*N* = 1784)

5,10-*MTHFR*	Smoking status	Overall (*N* = 1784)			Males (*N* = 873)			Females (*N* = 911)		
		
		*n*	Adjusted Mean birth weight (SE) g	Adjusted Δ B (SE) [95% CI] g	*n*	Adjusted Mean birth weight (SE) g	Adjusted Δ B (SE) [95% CI] g	*n*	Adjusted Mean birth weight (SE) g	Adjusted Δ B (SE) [95% CI] g
A1298C genotype										
AA	Nonsmoker	342	3071.9 (20.47)	Reference	169	3145.63 (28.99)	Reference	173	3000.74 (27.94)	Reference
	Passive smoker	589	3036.45 (15.32)	−29.04 (22.36) [−72.91, 14.82]	266	3069.39 (23.03)	−59.35 (31.34) [−120.86, 2.16]	323	3009.36 (20.43)	−3.07 (32.08) [−66.03, 59.90]
	Smoker	187	2973.15 (26.53)	−106.59 (30.12) [−165.67, −47.52]***	94	3045.5 (31.69)	−104.19 (41.36) [−185.37, −23.03]*	93	2900.02 (41.47)	−113.45 (44.05) [−199.91, −26.99]*
AC	Nonsmoker	182	3058.19 (29.74)	−1.66 (30.08) [−60.66, 57.34]	83	3110.86 (45.78)	13.56 (42.70) [−70.26, 97.37]	99	3015.1 (38.68)	−13.88 (42.68) [−97.64, 69.89]
	Passive smoker	319	3049.36 (20.36)	−15.20 (25.59) [−65.39, 34.98]	173	3066.73 (25.49)	−51.98 (34.39) [−119.47, 15.51]	146	3028.88 (32.66)	21.20 (38.41) [−54.19, 96.59]
	Smoker	90	3027.25 (42.41)	−57.04 (39.11) [−133.74, 19.66]	46	3120.85 (51.65)	−59.18 (53.47) [−164.14, 45.78]	44	2927.12 (65.41)	−64.21 (58.00) [−178.05, 49.62]
CC	Nonsmoker	31	2959.81 (55.2)	−120.92 (61.81) [−242.16, 0.32]^†^	17	3037.65 (79.96)	−107.17 (81.49) [−267.12, 52.77]	14	2865.29 (69.02)	−142.72 (94.43) [−328.05, 42.61]
	Passive smoker	38	3092.97 (65.72)	13.12 (61.81) [−97.08, 123.31]	20	3158.9 (98.97)	14.55 (75.56) [−133.75, 162.84]	18	3019.72 (84.17)	6.64 (84.21) [−158.64, 171.91]
	Smoker	6^c^	2976.17 (247.92)	—	5^c^	3054.6 (288.05)	—	1^c^	2584	—
	*P*_interaction_			0.04			0.03			0.22
A1298C allele^d^										
A	Nonsmoker	524	3069.05 (14.52)	Reference	252	3140.25 (20.45)	Reference	272	3004.92 (20.68)	Reference
	Passive smoker	908	3045.91 (10.93)	−15.55 (17.17) [−49.23, 18.12]	439	3075.06 (15.24)	−35.44 (24.30) [−83.13, 12.26]	469	3016.12 (15.67)	−6.65 (24.40) [−45.24, 50.54]
	Smoker	277	2978.20 (19.91)	−92.71 (26.38) [−144.46, −40.96]***	140	3043.18 (27.23)	−79.65 (36.21) [−150.73, −8.58]*	137	2912.57 (29.35)	−107.16 (38.53) [−182.78, −31.54]**
	*P*_interaction_			0.15			0.22			0.47
C	Nonsmoker	213	3049.58 (22.66)	Reference	100	3124.26 (32.36)	Reference	113	2980.20 (31.83)	Reference
	Passive smoker	357	3056.17 (17.48)	−18.49 (19.73) [−20.21, 57.20]	193	3084.82 (23.06)	−4.28 (26.42) [−56.13, 47.58]	164	3031.86 (26.57)	41.38 (29.65) [−16.81, 99.57]
	Smoker	97	3009.71 (33.78)	−29.12 (34.99) [−97.75, 39.50]	51	3070.73 (44.90)	−19.37 (47.02) [−111.66, 72.92]	45	2942.56 (51.53)	−46.16 (52.88) [−149.95, 57.64]
	*P*_interaction_			0.37			0.72			0.51

***P < 0.001. *P < 0.05. ^†^*P* < 0.1. Δ (Change in mean birth weight). B (Unstandardized coefficient) SE (Standard error). CI (Confidence interval), g (Grams). ^a^Multiple linear regression adjusted for infant sex, gestational age at delivery, maternal age, maternal height, prepregnancy weight, parity, alcohol intake during pregnancy, and folic acid supplement intake. ^b^Multiple linear regression adjusted for gestational age at delivery, maternal age, maternal height, prepregnancy weight, parity, alcohol intake during pregnancy, and folic acid supplement intake. ^c^Excluded from the regression analyses. ^d^*P*_trend_ by smoking status (A allele = 0.091, Males = 0.228, Females = 0.112 and C allele = 0.752, males = 0.828, Females = 0.271).

In cross-classification interactive analyses, infants born to nonsmokers with 5,10-*MTHFR* 677CT genotypes had the highest mean birth weight (3092 g); male newborns were 90.70 g (95% CI: 6.00 to 175.50, *P* = 0.036) heavier than the male infants of nonsmoking 677CC mothers, (*P*_interaction_ = 0.020; Table 3). The 5,10-*MTHFR* 1298AA genotype was associated with a 107.00-g (95% CI: −165.67 to −47.52, *P* < 0.001) decrease in mean infant birth weight among smokers. Stratification by infant sex did not yield obvious differences in birth weight, *P*_interaction_ = 0.040; Table 4). When 1298AC was set as the reference category, the 5,10-*MTHFR* 1298AA genotype was associated with a 107.00-g (95% CI, −180.00 to −33.90, *P* = 0.004) decrease in mean infant birth weight in smokers; the effect was more obvious in male infants (117.00 g; 95% CI: −218.60 to −14.70, *P* = 0.025; data not shown).

## DISCUSSION

Among nonsmokers, we found an association of maternal 5,10-*MTHFR* 677CT heterozygosity with higher infant birth weight, while 5,10-*MTHFR* 677TT homozygosity was associated with lower birth weight among female infants of passive tobacco smokers. In addition, among smokers, 5,10-*MTHFR* 1298AA homozygosity was associated with low folate status and lower birth weight. To our knowledge, this is the first study to report such findings for a Japanese population.

### Maternal 5,10-*MTHFR* C677T, *MTHFR* A1298C and serum folate status

Our results showed an association between the 5,10-*MTHFR* 677T allele and low folate status, which agrees with the findings of earlier reports.^6^^,^^46^ 677TT homozygosity was associated with low folate status, and values were much lower among active and passive smokers, which suggests independent and combined effects of tobacco smoke and 5,10-*MTHFR* C677T polymorphism on folate status.

In contrast, the 5,10-*MTHFR* 1298C allele was associated with higher serum folate levels, while the 1298AA genotype was associated with lower folate levels. Although the metabolic and clinical functions of this SNP have not been fully characterized, it is currently being studied by a number of investigators. In a recent study of Koreans, mean plasma homocysteine was higher among 1298AA homozygotes as compared with those carrying the 1298C allele.^48^ Because serum folate is inversely correlated with plasma homocysteine, we inferred that our study population might have a similar plasma homocysteine distribution across genotypes. A report from Portugal noted that 1298AC heterozygosity was associated with a high plasma folate level and that the level was lowest among 1298CC homozygotes,^49^ which is similar to the findings of the present study. Our findings contradict those of a study of a Dutch population, in which *MTHFR* A1298C alone was not associated with any biochemical abnormalities except when in combination with *MTHFR* C677T, specifically compound heterozygosity 5,10-*MTHFR* 677CT/1298AC.^28^ The fact that our findings were similar to those from a report on a Korean population is genetically plausible because racial, geographic, and nutritional disparities might account for differences in the functional characteristics of 5,10-*MTHFR* SNPs.^50^^,^^51^

### Effects of maternal 5,10-*MTHFR* A1298C polymorphism and tobacco smoke on infant birth weight

Smokers carrying 1298A alleles delivered infants with lower mean birth weights, especially female infants; however, these results must be interpreted with caution because alleles do not act in isolation. The effect of the maternal 5,10-*MTHFR* 1298AA genotype in reducing the birth weight of infants delivered by tobacco smokers might be due to low folate status associated with the 1298AA genotype. Perhaps some essential folate-dependent cellular processes were compromised. Cells that lack folate have been observed to accumulate in the S-phase of the cell cycle. Such cells have higher uracil misincorporation and DNA damage,^52^ which might have a role in the impairment of fetal growth. Moreover, chronic deficits in extracellular and intracellular folate due to the effects of tobacco smoke might have been severe enough to inflict nutritional stress. In our study, we could not examine the role of the 1298CC genotype among smokers because of its low frequency. However, previous reports observed that the maternal 1298CC genotype was associated with a greater reduction in the risk of low birth weight as compared with the 1298AA genotype.^21^ The 1298CC homozygous genotype was also reported to be protective against IUGR in Canadians.^53^ Hyperhomocysteinemia might have increased the risk of placental vasculopathy via oxidative stress, endothelial cell dysfunction, and/or coagulopathies leading to feto-placental hypoperfusion.^5^

Adequate serum vitamin B_12_ status has been shown to decrease total plasma homocysteine levels in Japanese.^54^ However, among smokers, the possible coexistence of nutritional deficiencies, including vitamin B_12_ deficiency, might have compromised the methylation of homocysteine to methionine, resulting in impaired fetal growth. The folate level was probably not adequate to silence the phenotypic expression of 1298AA among smokers. Higher exogenous folate may be needed to correct deficits and maintain ideal levels for optimal fetal growth.

With regard to the 5,10-*MTHFR* gene structure, the A1298C variant is located on the regulatory C-terminal domain, which contains protein retention signals that prevent delivery of proteins to the secretory pathway. It is possible that allosteric inhibitory interplay in the s-adenosyl methionine (SAM) and s-adenosyl homocysteine (SAH) cycle is involved in the functional behavior of this SNP in relation to folate status and mediation of fetal growth. Recently, the 5,10-*MTHFR* A1298C polymorphism was found to be associated with increased folate levels in red blood cells, in an inverse relationship with 5,10-*MTHFR* C677T polymorphism,^55^ which suggests that both SNPs have different functional characteristics with regard to phenotypic expression.

Due to limited evidence on the functional role of the 5,10-*MTHFR* A1298C polymorphism, especially among Japanese, further research is needed to verify this observation and elucidate the biological mechanisms associated with this SNP.

### Effects of the maternal 5,10-*MTHFR* C677T polymorphism and tobacco smoke on infant birth weight

The 5,10-*MTHFR* 677T allele is associated with low folate and high homocysteine levels. In this study, the 677T allele was associated with lower birth weight in offspring of active smokers. The 677CT genotype was protective against low birth weight, especially among male offspring, but only in the absence of active or passive tobacco smoke. This might be due to the presence of higher mean serum folate levels among nonsmokers. In a Korean population, the 677T allele had a weak protective association with lung carcinoma.^56^ The protective effect of adequate folate status might be mediated by the stabilization of flavin-adenine-dinucleotide (FAD) binding at the catalytic domain.^2^ The birth weight of female infants of 677TT homozygous passive smokers was significantly lower than that of female infants born to passive smokers of similar genotypes. Male fetuses were favored, probably because pregnant mothers carrying male fetuses have a higher nutritional intake than those with female fetuses.^57^ Poorly understood phenomena on fetal sex-specific signals have been implicated in fetal growth, especially in response to glucocorticoid activity that might modify the fetal response to stress.^58^

### Study strengths and limitations

The participants were indigenous Japanese; hence, we overcame issues of population stratification in genetic association studies. Overall, this study might be limited by selection bias, as we utilized integrated data from only 61% of the total recruitment during the study period. However, because we randomly selected the final study population based on known pooled population frequencies of the genetic factors and tobacco smoking among pregnant women, we believe that this study does not substantially differ from one with a higher participation rate (ie, ≥70%). The sample size of the study was adequate to detect gene-environment interactions; however, multiple comparisons and small sub-group sample sizes might have affected the study power. There could be misclassification bias from self-reported tobacco smoking; however, a previous study reported very low misclassification bias among Japanese women.^59^ Therefore, the findings of the present study are likely to be reliable. Nevertheless, this does not diminish the importance of using biomarkers like cotinine. The findings might have been confounded by other B vitamins, which were not studied, or by other unidentified sources common to cohort study designs. Finally, this study was hospital-based; therefore, our findings should not be generalized. Further studies of other factors in the folate-homocysteine pathway should prove interesting.

### Public health implications

Analysis of the 5,10-*MTHFR* A1298C polymorphism shows that the frequency of the 1298AA genotype is greater than 60.0% in the Japanese population.^38^ Its association with low folate status is thus a considerable public health concern. With the recent increasing prevalence of smoking among young Japanese women, particularly in Hokkaido,^60^ maternofetal morbidity and mortality might also increase. Smoking cessation and targeted use of folic acid supplements could therefore prove to be very important public health tools in this population.

### Conclusions

Our findings suggest that the maternal 5,10-*MTHFR* C677T polymorphism is independently associated with higher infant birth weight, especially among nonsmokers, while the 5,10-*MTHFR* A1298C variant is not independently associated with birth weight. In addition, the 5,10-*MTHFR* 1298AA polymorphism might be associated with folate impairment and could interact with tobacco smoke to further decrease birth weight.
